# Review of "Immunological Bioinformatics" by Lund, Nielsen, Lundegaard, Kesmir & Brunak

**DOI:** 10.1186/1475-925X-5-32

**Published:** 2006-05-18

**Authors:** Andrew Lloyd

**Affiliations:** 1 Education & Research Centre, St. Vincent's University Hospital, Dublin, Ireland

## 

Since the late 80s of the last century, I've been earning a crust teaching and researching in the field of bioinformatics and molecular evolution. Recently, I have tentatively and with trepidation crossed the fence between that familiar territory and the rolling unknown prairies of immunology. It is frightening out there: immune cells are named for but one of the molecules that they express; molecules with similar names have no phylogenetic relationship; the same molecule will have different names; and the subtleties of self and non-self have me in an existential crisis. So I was delighted to get this book. I thought that it would give an overview of my new cross-disciplinary field of interest, suggest all sorts of avenues to investigate, point me to the relevant tools, give some background to their effective application and empower me to stride confidently forward. Immunology can be viewed, as the authors point out, as being as big as the rest of biology put together; you cannot cover everything in 300 pages and we must allow editors and authors discretion to cover what they know best or what they feel is most important. But it is idiosyncratic to give as much space to the analysis of DNA microarrays (a technique that any molecular biologist or immunologist would consider using and which requires a good level of statistical competence to do effectively) as to explaining the mechanics of alignment by dynamic programming (which could easily be left comfortably within the black box of whatever alignment tools that same biologist uses to compare sequences). And sadly, my own area of particular interest, the whole immunological dark side of innate immunity, is essential untouched.

On the other hand, there is a workmanlike introduction to information content in sequences, how this is calculated, how such data can be represented and how sample size impinges critically on the accuracy of estimates. That chapter moves on to explain, at an appropriate and informative level of detail, Gibbs Sampling, Hidden Markov Models and Artificial Neural Networks. Much of the rest of the book looks at how to predict and classify epitopes and other sequence recognition motifs and the book is illustrated with hundreds of Logo representations of this variability. The final chapter draws the rest together to describe an integrated approach for using bioinformatic tools to predict antigenicity. Wherever possible, an explicit connexion is made between the theory and algorithms and the research that has been carried out using these techniques on real, and important, biomedical problems. Accordingly, this is a good overview of how computational techniques can inform our understanding of adaptive immunity.

